# Cutaneous Infections Caused by *Trichophyton indotineae*: Case Series and Systematic Review

**DOI:** 10.3390/jcm14041280

**Published:** 2025-02-14

**Authors:** Aurora De Marco, Giovanni Liguori, Claudia Cafarchia, Francesco Triggiano, Giulia Ciccarese, Melita Anna Poli, Francesca Ambrogio, Domenico Bonamonte, Nicoletta Cassano, Gino Antonio Vena, Caterina Foti, Giuseppina Caggiano

**Affiliations:** 1 Section of Dermatology and Venereology, Department of Precision and Regenerative Medicine and Ionian Area (DiMePRe-J), University of Bari “Aldo Moro”, Piazza G. Cesare 11, 70124 Bari, Italy; aurorademarco94@gmail.com (A.D.M.); giovanni.liguori@policlinico.ba.it (G.L.); m.poli94@gmail.com (M.A.P.); dottambrogiofrancesca@gmail.com (F.A.); domenico.bonamonte@uniba.it (D.B.); caterina.foti@uniba.it (C.F.); 2 Dipartimento di Medicina Veterinaria, Università degli Studi di Bari, Str. prov. per Casamassima Km 3, Valenzano, 70010 Bari, Italy; claudia.cafarchia@uniba.it; 3 Section of Hygiene, Interdisciplinary Department of Medicine, University of Bari “Aldo Moro”, Piazza G. Cesare 11, 70124 Bari, Italy; francesco.triggiano@uniba.it (F.T.); giuseppina.caggiano@uniba.it (G.C.); 4 Section of Dermatology, Department of Medical and Surgical Sciences, University of Foggia, Viale Pinto 1, 71122 Foggia, Italy; 5 Private Practice in Dermatology and Venereology, 76121 Barletta, Italy; nicoletta.cassano@yahoo.com (N.C.); ginovena@gmail.com (G.A.V.)

**Keywords:** infectious diseases, dermatophytosis, fungal infections, tinea, *Trichophyton indotineae*

## Abstract

**Background/Objectives**: Dermatophytosis due to *Trichophyton* (*T.*) *indotineae* has spread worldwide, and the acquisition of new drug resistances is making this threat challenging to face. We report four cases of dermatophytosis caused by *T. indotineae* and perform a systematic review of case reports to explore the most relevant clinical and demographic features and the treatment patterns of this infection. **Methods**: A literature search, using the PubMed database and following PRISMA guidelines, was performed up to the 6th of December 2024. Articles were included if written in English and presented in the form of case reports/series involving human subjects, with detailed information and *T. indotineae* infection confirmed by internal transcribed spacer sequencing. **Results**: Initially, 255 records were identified, and 30 articles were finally selected, including 64 patients, mainly from the Asian continent. Most patients were healthy and/or immunocompetent (65.52%), and the mean disease duration suggested long-lasting lesions. At least two different body sites were generally involved, with a predilection for lower body areas (groins included), as also observed in our patients (all from South Asia). Review results indicated itraconazole as the most commonly prescribed final medication. Treatment with itraconazole led to complete remission in three of our patients (one patient was lost to follow-up). **Conclusions**: *T. indotineae* infection should be suspected in case of extensive and/or recalcitrant dermatophytosis, especially in patients with a travel history to Asian countries. Further research is needed to develop rapid, inexpensive, and accurate techniques for the identification of *T. indotineae* and drug-resistant strains and to define the optimal preventive and treatment strategies.

## 1. Introduction

Dermatophytoses are superficial fungal infections caused by filamentous and keratin-degrading fungi known as dermatophytes [1]. This infection involves humans and animals, affecting skin, hair, and nails, with only rare cases of disseminated and invasive infections, especially in immunocompromised patients [1]. Dermatophytes are classified depending on their habitat into anthropophilic, zoophilic, and geophilic and various genera (e.g., Trichophyton, *Microsporum*, and *Epidermophyton*), accounting for over 40 species known to infect humans [2]. The clinical condition resulting from a dermatophyte infection and its associated inflammatory response is called “tinea”. It may involve different anatomical sites, thus leading to classification into specific variants (tinea corporis, tinea manuum, tinea pedis, tinea cruris, tinea capitis, tinea faciei, tinea barbae, and tinea unguium) [2].

Dermatophytosis affects approximately 25% of the global population, although its incidence may reach 60% in African and Asian countries [1]. *Trichophyton (T.) rubrum, T. mentagrophytes* species complex, *Microsporum canis*, and *Epidermophyton floccosum* are the most prevalent causative agents worldwide [3]. However, the epidemiology of dermatophytosis changes continuously due to tourism and migration, and new genera have recently been added [3].

Interest in dermatophytosis is increasing due to its widespread presence and rising antifungal resistance. Initially, in the early 1980s–1990s, resistance to antifungal treatments was described for griseofulvin, mainly in the case of tinea corporis caused by *T. rubrum*. Recently, alarming outbreaks of new resistance to oral terbinafine and other antifungals have emerged worldwide, especially in the Indian subcontinent [3]. In this regard, between 2015 and 2017, the first cases of an unusual strain of *T. inderdigitale*, highly resistant to terbinafine, were reported in India, followed by the isolation of similar strains in other Asian countries [3,4]. Therefore, a newly defined entity was described among dermatophytes previously referred to as *T. mentagrophytes* genotype VIII and finally *T. indotineae* [3,4]. As morphological differences between *T. indotineae*, *T. mentagrophytes*, and *T. interdigitale* are minimal, these species cannot be distinguished by traditional methods. *T. indotineae* can indeed be differentiated by specific genetic sequences, so it gained full recognition as an individual species in 2020 [3]. In fact, sequencing of the internal transcribed spacer (ITS) region of ribosomal DNA is currently the most accurate method for the identification of *T. indotineae* [5]. *T. indotineae* frequently displays resistance to terbinafine, mainly due to mutations in the gene encoding for squalene epoxidase (SQLE), a central enzyme in the synthesis of ergosterol [3].

For this reason, the global spread of *T. indotineae* infection may lead to new therapeutic challenges. Moreover, although dermatophytoses due to *T. indotineae* were originally confined to the Indian subcontinent, they have been increasingly reported in other countries worldwide, mainly attributed to travel and migration [4,5]. However, cases of possible local acquisition and transmission without any apparent connection with previously identified patients and with no history of travel to endemic countries suggest the potential risk for untraceable outbreaks [5,6].

The present article describes four documented cases of skin infection caused by *T. indotineae* in Southern Italy and systematically reviews and summarizes the case reports available in the current literature to highlight clinical and therapeutic aspects that can help clinicians manage this infection.

## 2. Relevant Sections

### 2.1. Case Reports

#### 2.1.1. Patient 1

An otherwise healthy 36-year-old woman from Bangladesh who has been living in Italy for about 10 years with her husband presented with a 6-month history of diffuse itching dermatosis. Of note, a few weeks before the onset of the rash, she was visited by her brother from Bangladesh, who had similar lesions on his groin. History did not reveal contact with animals.

**Figure 1:**
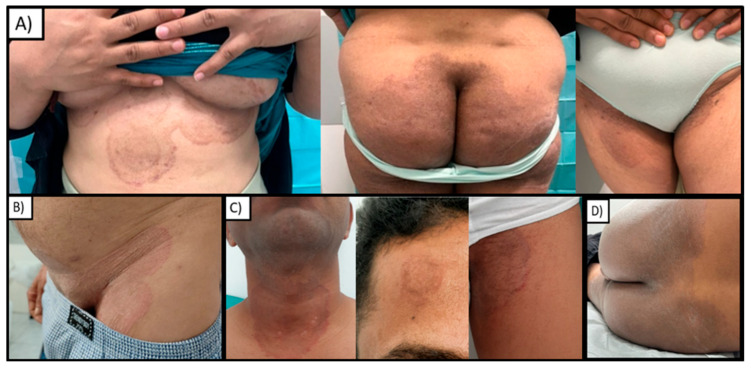
(**A**) Patient 1: annular and figurate patches on inframammary folds, with a double-edged scaly border and a concentric distribution; less defined patches with hyperpigmentation on the buttocks; erythematous patches with slight hyperpigmentation on inguinal folds. (**B**) Patient 2: well-demarcated, erythematous, and scaly patches affecting the lower abdomen, pubic area, and groins. (**C**) Patient 3: a large erythematous-violaceous circular patch on the anterior neck; a smaller similar patch on the forehead; erythematous and scaly patches with raised borders on the groin. (**D**) Patient 4: diffuse coalescing scaly lesions with prominent pigmentation on the buttocks.

Physical examination showed well-demarcated, mildly erythematous, and scaly patches with occasional vesicles and pustules on her abdomen, groins, buttocks, and inframammary folds (Figure 1A). A figurate pattern was evident on the inframammary folds with a double-edged border and a concentric distribution. Lesions coalesced in a more irregular pattern on other areas, especially the buttocks, with less defined patches and hyperpigmentation.

Dermatophytosis was suspected, and skin scraping samples were collected for fungal culture, which identified *Trichophyton* spp. Therapy with oral terbinafine (250 mg/day) and ciclopirox olamine 1% cream was prescribed. However, after one month of therapy, there was no clinical improvement, so resistance to terbinafine was suspected. For this reason, an antifungal susceptibility testing was performed, documenting sensitivity to itraconazole. Molecular investigations were carried out to identify the culprit species, thus revealing the presence of a *T. indotineae* strain. Treatment with oral itraconazole (200 mg/day for 4 weeks and then 100 mg/day for the other 4 weeks) led to complete clinical remission and was well tolerated.

#### 2.1.2. Patient 2

After visiting patient 1, she asked us to examine her husband as well, as he recently noted similar pruritic skin lesions on his abdomen. Patient 2 was an otherwise healthy 40-year-old man from Bangladesh, denying any concomitant systemic treatments as well as any contact with animals.

Physical examination revealed the presence of well-demarcated, erythematous, and scaly patches affecting the lower abdomen, pubic area, and groins (Figure 1B). Also in this patient, species identification techniques, including molecular analysis, confirmed an infection caused by *T. indotineae*, so a possible sexual transmission was hypothesized. Treatment with oral itraconazole (200 mg/day) resulted in complete clinical remission after 4 weeks without any adverse events.

#### 2.1.3. Patient 3

The third patient was a 49-year-old male from Sri Lanka who reported the onset of mildly pruritic skin lesions several months before consultation during a trip back to his original country. He did not report any concomitant diseases and systemic treatments, as well as any contact with animals or infected subjects.

Well-demarcated, annular, erythematous, and scaly patches were present on the anterior part of his neck and also on his forehead and groins (Figure 1C). A culture-identified *Trichophyton* spp. Due to the spread of the lesions and the recent travel to an endemic area, molecular identification of fungal species was carried out, leading to *T. indotineae* recognition. Complete resolution was achieved after treatment with oral itraconazole (100 mg twice a day for 4 weeks, then 100 mg/day for 4 weeks). No adverse reactions were observed.

#### 2.1.4. Patient 4

A healthy 33-year-old male patient from India showed an itching rash that began about six months earlier during a trip to India. He reported previous use of different topical medications, such as antifungal agents and corticosteroids, without clinical improvement. However, difficulties in collecting other information regarding history and possible sources of infection were encountered due to linguistic barriers. Physical examination revealed erythematous patches with slight desquamation and prominent hyperpigmentation involving his lower limbs, abdomen, buttocks, and inguinal folds (Figure 1D). A *Trichophyton* spp. infection was demonstrated by conventional mycology techniques, thus increasing suspicion of *T. indotineae*, which was subsequently confirmed by molecular investigations. The patient was started on oral itraconazole (100 mg twice a day), but he was lost to follow-up.

### 2.2. Identification of Isolates and Antifungal Susceptibility Testing

For each patient, cutaneous scarification was performed with a sterile blade. All samples were individually packaged, and standard laboratory disinfection and decontamination procedures were followed to minimize the possibility of cross-contamination.

Samples were cultured on 90 mm Petri dishes containing Mycobios Selective Agar (Biolife italiana srl, Monza, Italy) and Sabouraud dextrose agar (SDA) with chloramphenicol (Biolife italiana srl) and incubated at 24–30 °C, respectively, for 2–4 weeks and examined every 2 days. Fungal colonies grown in the medium were examined by macroscopic and microscopic observation of the hyphae, macroconidia, and microconidia.

Fungal strains were molecularly confirmed by amplification (with polymerase chain reaction, PCR) and nuclear ribosomal ITS region sequencing. Genomic DNA was isolated from each sample using the DNeasy Blood Tissue Kit [QIAGEN, Hilden, Germany], following the manufacturer’s instructions. The nuclear ribosomal ITS region was amplified using ITS1 (5′-TCCGTAGGTGAACCTGCGG-3′) and ITS4 (5′-TCCTCCGCTTATTGATATGC-3′) primers.

PCR products were examined on a 2% agarose gel stained with GelRed (VWR International PBI, Milan, Italy) and visualized on a ChemiDoc Imaging System (Bio-Rad Laboratories, Inc., Segrate, Italy). PCR products were enzymatically purified using the ExoSAPT-IT reagent (Thermo Fisher Scientific, Segrate, Italy) and bidirectionally sequenced at Eurofins Genomics (Ebersberg, Germany) using standard Sanger sequencing and the same primer set used for PCR.

Raw nucleotide sequences were edited using the FinchTV v1.4 software (GeoSpiza Inc., Seattle, WA, USA). For each genetic marker, the final DNA consensus sequence was generated by matching both forward and reverse sequencing traces and then extracted in a text-based FASTA format, which was used for the taxonomic recognition of the fungal isolate by Basic Local Alignment Search Tool (BLAST, National Center for Biotechnology Information, Bethesda, MD, USA; http://blast.ncbi.nlm.nih.gov/Blast.cgi, 15 January 2025) of similar sequences deposited in the GenBank (http://www.ncbi.nlm.nih.gov/genbank, 15 January 2025).

Antifungal susceptibility was evaluated for the *T. indotineae* isolate by E-test performed as recommended by the manufacturer (AB BIODISK, Solna, Sweden). The minimum inhibitory concentration (MIC) was read as the drug concentration at which the boundary of the elliptical inhibition zone intercepted the MIC scale on the E-test strip.

Dermatophytes were isolated from all four samples. The macro- and microscopic identification of the isolated strains confirms the isolation of *Trichophyton* spp., which was molecularly identified as T. indoline. All four sequence types matched with previously published sequences (MN460830.1; MN460831.1.; MN460832.1; MN460833.1; and OR416997.1) with a different % of identification and query cover (Table 1). In particular, two ITS sequence types, herein named ITSa and ITSb, were identified using the Basic Local Alignment Search Tool (Table 1).

In general, all four dermatophyte strains showed similar patterns of susceptibility to each antifungal agent tested, with high MIC values found for fluconazole, amphotericin B, and caspofungin. More specifically, the in vitro susceptibilities by the E-test method showed the following MIC ranges (μg/mL^−1^): fluconazole > 256; amphotericin B 2–8; caspofungin 2–32; ketoconazole 0.002–0.008; anidulafungin 0.008–0.012; voriconazole 0.002–0.004; micafungin 0.25; itraconazole 0.047.

Susceptibility to terbinafine was only clinically demonstrated and not laboratory assessed, as it was not included in our hospital testing panel.

### 2.3. Systematic Review of Case Reports

#### 2.3.1. Methods

A literature search, using the PubMed database and following the Preferred Reporting Items for Systematic Reviews and Meta-Analyses (PRISMA) guidelines, was carried out, including all records up to the 6th of December 2024 [7]. Only articles in English were selected. The search strategy included the following keywords: [*Trichophyton indotineae*], [*Trichophyton indotineae*] AND [diagnosis], [*Trichoptyhon indotineae*] AND [management], [*Trichophyton indotineae*] AND [treatment]. The systematic review was not recorded in the register.

The present review aimed to highlight epidemiological and clinical data regarding *T. indotineae* skin infection, focusing on management. For this reason, to offer a more practical point of view, this review has included case reports and case series currently available in the literature. Therefore, to be included in this systematic review, articles had to meet the following inclusion criteria: i. articles written in English; ii. articles involving human subjects; iii. articles presented in the form of case reports and case series; iv. articles containing detailed data on every single patient, e.g., general characteristics, clinical features, diagnosis, and therapeutic management of the infection; and v. infection by *T. indotineae* confirmed by molecular analysis [ITS genome sequencing]. Initially, two reviewers carried out an independent screening of titles and abstracts. Afterward, the full-text examination was performed to assess the fulfillment of inclusion criteria. Therefore, in case of discrepancies, a third author was involved.

Data were collected using a standardized template by two independent reviewers. The extracted data included patients’ data (gender and age), epidemiological data (contact with possibly infected patients, geographical localization and origin, travel history), clinical data (involved body sites, disease duration, medical history), tools for diagnosis (e.g., gene sequencing), previously failed treatments, and final treatment (drug, dosage, duration, outcome). Any discrepancies were resolved by a third author throughout deliberation.

The Joanna Briggs Institute (JBI) was used to determine the risk bias assessments. Specific versions were used for both case reports and case series [8]. Any discrepancies in this process were solved through consensus after deliberations among authors.

#### 2.3.2. Results

**Figure 2:**
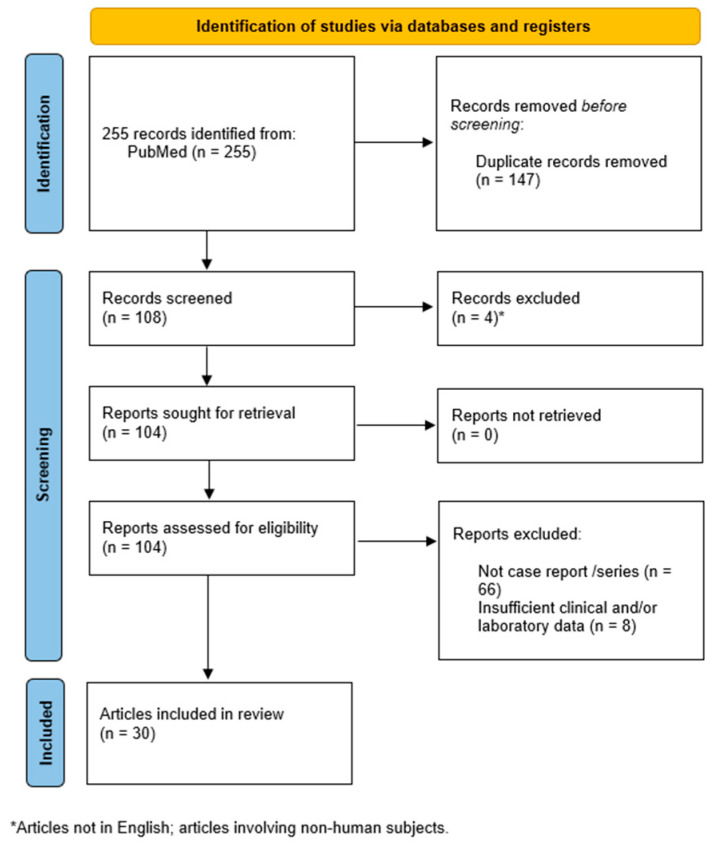
Flowchart illustrating the selection of studies included in this review, according to the Preferred Reporting Items for Systematic Reviews and Meta-Analyses (PRISMA) guidelines [39].

A systematic search initially identified 255 records. After excluding duplicates, 108 articles were reviewed. During the screening process, four articles (non-English publications and/or studies evaluating non-human subjects) were eliminated. Moreover, 74 articles were excluded as they did not contain detailed descriptions of case reports (n = 66) or showed insufficient clinical and/or laboratory data (n = 8). Therefore, 30 articles were finally selected for data collection [9,10,11,12,13,14,15,16,17,18,19,20,21,22,23,24,25,26,27,28,29,30,31,32,33,34,35,36,37,38] (Figure 2).

Supplementary Table S1 summarizes the demographic, epidemiological, clinical, and therapeutic data of the case reports included in the present review.

A total of 64 patients were therefore included in the present review, accounting for 33 males and 31 females. Age was not available for one patient out of 64, although in 13 cases, it was only generically assessed (e.g., <10 years, the 30s, 40s, etc.). The remaining patients’ ages ranged from 15 to 78 years, with an average of 36.

Geographic origin corresponded to different areas of the Asian continent in 37 out of 64 patients (57.81%), excluding 1 patient from the Middle East. India (18 cases) and Bangladesh (7 cases) were the predominant countries. However, even when geographical origin was not specified (22 cases out of 64), a significant history of travel to an Asian country was reported in 14 cases.

Data regarding transmission routes were accessible for 35 patients out of 64, including 30 patients with clear information regarding their family members, 4 patients with data concerning possible sources of contamination, and 1 patient with both types of information. Infection in the household/close contacts was documented in 14 cases [9,13,17,21,25,32]. At the same time, as for possible sources of contamination and behavioral risk factors, two patients were healthcare workers (one nurse in a dermatology unit [15] and one medical doctor in an emergency department [16]), one patient had a history of a possible sexual transmission [35], and two subjects reported the common habit of sharing clothes, towels, or wetsuits [23,36]. Moreover, most articles did not include information regarding animal contact, as it was specified only for 8 patients out of 64, and 6 denied any contact.

Medical history was reported in 58 cases out of 64, and, when available, it documented relevant clinical data, possibly affecting patients’ immune response, in 7 patients out of 58 (12.07%) (4 patients with diabetes mellitus, 1 patient with altered levels of glycated hemoglobin, and 2 patients affected by hematological malignancies). Moreover, none of the screened patients tested positive for HIV and HTLV-1 infection (5 out of 58 and 2 out of 58, respectively). Most patients were reported to be healthy and/or immunocompetent (38 out of 58; 65.52%), while 9 patients out of 58 (15.52%) were affected by other medical conditions, not impacting the immune system. Of note, four women were pregnant, and one woman was breastfeeding.

Disease duration was considered as the period from “onset to confirmation” in 11 cases [25] and as the period from rash onset to clinical evaluation in the remaining patients. In contrast, it was not available for 15 patients and was not clearly expressed in six cases (e.g., “onset during summer”, “several months”, “>24 months”, etc.). On the other hand, considering reports with specified data, the mean duration was 11.82 months (range: 1.25–60 months).

As for clinical presentation, a single area (right leg, forearms, buttocks, and face, respectively) was affected in only four patients, while at least two different body sites were involved in all the remaining cases. Of note, three patients presented with a widespread infection, affecting most of the body surface [21,22,29].

Among the selected patients (with the exception of patients with generalized skin involvement), numerous sites of involvement were reported, with lower limbs (reported 36 times), groins (n = 34), buttocks (n = 29), and trunk (considered as chest, back, abdomen, and hips without folds and buttocks) (n = 29) being the most frequently reported areas. Upper limbs, pubic area, and face were affected in 17, 14, and 12 patients, respectively. In addition, no distinction between upper and lower limbs was possible in three cases, as the site of infection was generally referred to as “limbs”. Axillary pits, inframammary folds, and neck appeared to be only marginally involved, and hands and feet were rarely affected.

As for previous treatments, topical antifungals have been used by 41 patients, whereas 33 patients have applied topical corticosteroids alone or in combination with antifungal agents.

On the other hand, among prior oral treatments, oral terbinafine was the most frequently used. A total of 39 patients unsuccessfully received therapy with oral terbinafine, with only two cases of partial response. In 21 of these 39 patients, multiple courses and/or continuous treatments with a daily dose of 250 mg for more than 4 weeks were administered. Other previous oral medications included itraconazole (n = 15), fluconazole (n = 11), and griseofulvin (n = 9), which were mostly ineffective (a partial response was obtained with fluconazole in one patient and with itraconazole in another patient, whereas a clinical remission followed by relapse after treatment discontinuation was observed with itraconazole in two subjects and with fluconazole in one patient).

As for the final treatments, itraconazole was the most commonly prescribed medication. It was administered in 36 patients with variable dosages (most frequently 200 mg/day) and duration as monotherapy or combined with different topical antifungal drugs (10 patients). Two patients discontinued itraconazole treatment due to adverse events, and one patient was lost to follow-up. Improvement or remission was obtained with itraconazole in 32 patients (89%); however, after treatment discontinuation, 8 of these 32 patients had recurrences, often requiring retreatment with itraconazole. Other final treatments included oral terbinafine (four patients; remission in two), oral fluconazole (six patients; remission in two), and oral griseofulvin (six patients; remission in one). Notably, griseofulvin liquid was erroneously used topically instead of orally by a patient who surprisingly obtained positive effects [27]. Only four patients were treated with topical therapy.

Oral voriconazole, mentioned in previous treatments for two patients with presumably poor results [25], was successfully used in three cases [15,32], whereas posaconazole was found to be effective in four patients [30]. Interestingly, a case report suggested the possible use of topical voriconazole [10].

## 3. Discussion

In the past decades, due to migration and tourism with subsequent local transmission, dermatophytosis due to *T. indotineae* has spread worldwide, involving not only the Indian subcontinent but also many other areas outside the endemic ones, including Europe and North America [40]. However, the prevalence of *T. indotineae* infection might be underestimated as traditional methods are unable to distinguish *T. indotineae* from *T. mentagrophytes* and *T. interdigitale* [41].

As for laboratory confirmation of a suspected *T. indotineae* infection, indeed, although classical mycological techniques may be helpful in patients’ follow-up assessment, definitive diagnosis requires molecular-based techniques, which may not be available in many routine laboratories [42]. Therefore, sequencing of the ITS region of ribosomal DNA is currently considered the gold standard for *T. indotineae* identification [5,40].

Moreover, increasing treatment failures, together with the acquisition of new drug resistances, have been reported. Besides resistance to terbinafine, with rates ranging from 17% to 75% in India, *T. indotineae* seems to display resistance to other antifungal agents as well, such as azole compounds [40,43,44]. Up to 25% of *T. indotineae* strains in India are less susceptible to itraconazole and voriconazole [44]. Mutations affecting the genes encoding for sterol 14-α demethylase, as well as their overexpression, have been implicated among the main mechanisms responsible for azole resistance, and an important role may be played by the overexpression of the *CYP51B* gene [5,43,44].

Therefore, early recognition of possible *T. indotineae* infections is becoming essential to prevent outbreaks from expanding and promptly activate a correct diagnostic and therapeutic protocol (Table 2) [4,25,42].

From a clinical point of view, *T. indotineae* infection generally presents in the form of tinea corporis and/or cruris and/or (less frequently) faciei, often characterized by scaly plaques with active borders, rapidly affecting multiple body sites with strong involvement of the lower body, including groins and buttocks [4,12,41,45,46]. Tinea manuum, tinea barbae, tinea pedis, and tinea unguium appear to be less common [4,47].

The results of our review seem to confirm both the predilection for the lower part of the body, as also observed in our four patients, and the tendency towards localization in multiple sites. One of our patients also had lesions on the forehead and neck.

All our patients complained of pruritus. The association with itching or burning sensation and the prolonged evolution over time are common features of *T. indotineae* infection, and the disease can be chronic and relapsing despite treatment [4,45,47]. Pigmentation can be seen, especially in patients with darker skin [42], and it was particularly evident in two of our patients (patients 1 and 4). The absence of central clearing within the affected areas is not unusual, according to Khurana et al. [42], and this finding was also observed in some of our patients. Skin lesions are often extensive [42,47], and have been described as highly inflammatory [4,45], sometimes with eczema-like features [6,42], although other authors have pointed out that lesions can present with only minimal localized scaling and negligible or absent inflammation [42]. The variable degree of inflammation could also be explained by applying topical corticosteroids [47], which were included among previous therapies in about half of the patients in our review. In this regard, steroid misuse and the abuse of topical steroids, often in combination with antimycotic or antibiotic agents in over-the-counter products, have been implicated as a risk factor for the emergence of antifungal resistance [46,47]. Immunosuppression and diabetes mellitus do not appear to have a relevant role as risk factors for *T. indotineae* infection, as these comorbid conditions were detected in a minority of cases [25,47], as also shown in our review. Intrafamilial transmission with easy spread is frequently observed, and the role of indirect transmission through fomites has been hypothesized [42,47]. Sexual transmission is also possible [35].

Species identification and antifungal susceptibility testing are essential to monitor mycological and drug susceptibility trends and support the choice of optimal therapeutic strategies [42]. However, antifungal susceptibility testing is a complex and time-consuming technique that is not routinely performed. Moreover, the lack of precise clinical breakpoints for dermatophytes has led to difficulties in interpreting testing results [5,41,42]. Nevertheless, validated tentative epidemiological cut-off values against *T. indotineae* using the European Committee on Antimicrobial Susceptibility Testing (EUCAST) protocol have been proposed [48,49]. In addition to antifungal susceptibility testing, evaluating mutations in the *SQLE* gene may indicate terbinafine resistance [5].

In a review concerning terbinafine-resistant *T. indotineae* in Europe [41], antifungal susceptibility testing provided a great range of MICs for terbinafine. It demonstrated that higher terbinafine MICs may be associated with mutations in the *SQLE* gene. On the other hand, in a case series of 11 patients with *T. indotineae* infection in New York City [25], results of antifungal susceptibility testing and *SQLE* gene variants exhibited an association with clinical resistance to terbinafine, whereas patients’ response to other antifungals did not correlate with MIC values. Nonetheless, other authors emphasized that higher MICs do not necessarily reflect clinical unresponsiveness, and some reports even documented failure to terbinafine therapy in patients with *T. indotineae* infection, despite low MIC values and the absence of mutations in the isolate [5,42].

Therefore, additional techniques are currently being explored in order to simplify and speed up *T. indotineae* identification and/or recognition of terbinafine-resistant strains, such as the Matrix Assisted Laser Desorption/Ionization Time of Flight Mass Spectrometry (MALDI-TOF MS) identification tool or the DermaGenius^®^ Resistance (Bruker Corporation, Billerica, MA, USA) real-time PCR assay for detection of terbinafine-resistant *Trichophyton* isolates [50,51,52]. Moreover, in order to detect more efficiently terbinafine-resistant strains, a certain utility of the terbinafine-containing agar method (TCAM) has been shown [53]. Recent data have also demonstrated that SYBR Green real-time PCR assays can be considered a rapid and accurate tool for identifying *T. indotineae* from clinical isolates as well [54].

As for therapeutic management, oral itraconazole is the most effective drug against *T. indotineae* and is currently considered the best treatment choice, especially if resistance to terbinafine is suspected and/or confirmed [42,55]. The most frequently administered dosage is 100–200 mg/day for a variable duration of 1 to 12 weeks, displaying a potential synergistic effect with topical antifungals [42,46,56]. Khurana et al. reported that a daily dosage of 100 mg can be sufficient in many cases and suggested continuing treatment until a cure, thus requiring a treatment period of at least 6–8 weeks in most patients [42]. Song et al. recommended oral itraconazole 200 mg/day for 4–8 weeks as the best treatment for terbinafine-resistant *T. indotineae* infection [57].

On the other hand, oral terbinafine (250 mg/day) is often used as a first-line treatment and can be effective in infections caused by *T. indotineae* strains sensitive to terbinafine [47]. Although more evidence-based data are needed, terbinafine 250 mg twice daily is considered a possible therapeutic option even in the case of terbinafine resistance, as higher dosages could counterbalance the effect of *SQLE* gene mutations and/or high MICs, at least in some patients [42]. However, the review by Sonego et al. showed the overall limited efficacy of oral terbinafine in treating *T. indotineae* infection [56].

Although the data are scanty, fluconazole and griseofulvin have limited in vitro or clinical activity [42,56]. It has been suggested that some patients may benefit from griseofulvin, probably using higher doses and long treatment durations [26]. In the case of multidrug-resistant strains, combination therapy with topical and systemic antifungals can be tried, and the off-label use of the newer triazoles may be considered [6].

The present systematic review confirms the preferential use and efficacy of itraconazole for the treatment of recalcitrant *T. indotineae* infection, as most patients experienced clinical improvement or remission. Unfortunately, relapses can occur after successful treatment, regardless of treatment regimens [42].

After the first observation of terbinafine resistance in the first patient of our series, consisting of four patients from South Asia, treatment with itraconazole was performed in all patients, with positive results achieved in three patients (one patient was lost to follow-up). *T. indotineae* infection has rarely been reported in Italy [14,58], and, to the best of our knowledge, this is the first report of cases of *T. indotineae* infection diagnosed in Southern Italy.

To obtain more practical information, we systematically reviewed cases of documented *T. indotineae* infection, focusing on reports with detailed information for each patient. Nevertheless, detailed data (e.g., regarding patients’ origin and travel history, medical history, clinical features, or past treatments) were not uniformly present in the examined reports. In addition, some patients’ outcomes were unknown because of pending follow-up. Moreover, short follow-up periods limit a reliable interpretation of therapeutic effects, also considering the possibility of recurrences after the completion of an effective treatment.

## 4. Conclusions

We documented *T. indotineae* infection in four patients who reported recent travels to endemic regions and/or contact with other affected subjects from those areas. One patient was lost to follow-up, whereas the other three adequately responded to oral itraconazole, which agreed with the literature data.

*T. indotineae* should be suspected in the case of extensive and/or recalcitrant dermatophytosis. A personal history of recent travel to endemic countries (e.g., South Asian countries), often in association with an inadequate response to conventional antifungal therapy, should increase suspicions.

Dermatophytosis due to *T. indotineae* has now spread worldwide. Moreover, the acquisition of new drug resistance is making this new threat difficult to face.

For these reasons, early recognition of possible *T. indotineae* infections is becoming essential, and the development of rapid, inexpensive, and accurate diagnostic techniques for the identification of *T. indotineae* and drug-resistant strains will be crucial.

Additional data are needed to define the optimal preventive and treatment strategies, and prolonged follow-up observations are necessary to obtain more precise data on the long-term response, given the risk of relapses after treatment discontinuation.

## 5. Future Directions

Finally, as antifungal resistance becomes increasingly alarming worldwide, innovative and multidisciplinary approaches will be necessary in the future to contain the problem and offer valid clinical solutions to treat multidrug-resistant infections [59].

Therefore, scientists, researchers, and clinicians should cooperate to establish a solid plan against antifungal resistance [59]. According to the World Health Organization, indeed, possible future goals focus on raising and spreading awareness regarding fungal diseases and resistance to common antifungal agents, addressing more efforts towards the development of new antifungal drugs, improving surveillance and diagnosis, possibly facilitating the recognition of resistant strains, and implementing targeted public health interventions globally [59].

## Figures and Tables

**Table 1:** *T. indotineae*: internal transcribed spacer (ITS) sequence types and percentage of sequence nucleotide identity with GenBank accession number.

Samples/Code Name	ITS Sequence Type	Nucleotide Identity (%)	Query Cover	Accession Number
Case 1/IG0a	ITSa	100%	100%	MN460830.1; MN460831.1.; MN460832.1; MN460833.1; OR416997.1
Case 2/IG0b	ITSa	100%	100%	
Case 3/IG1	ITSa	100%	100%	
Case 4/IG2	ITSb	99.84%	99%	

**Table 2:** Clues for suspecting infection caused by *T. indotineae*.

A long-lasting, relapsing, or recurrent itching dermatosis
Recent travel to a region with a high prevalence of *T. indotineae* infection or contact with a person who recently traveled to an endemic area (e.g., Asian countries, especially South Asia)
Evidence of diffuse skin lesions suggestive of tinea corporis (mainly when affecting the lower part of the body) and/or tinea cruris and/or (less commonly) tinea faciei
Prior culture identification of *T. mentagrophytes* or *T. interdigitale* in patients with compatible clinical findings and medical history
Recalcitrant dermatophytosis with inadequate response to topical antifungals and/or to oral antifungal medications (in particular oral terbinafine), used at standard doses and durations

## Data Availability

The data presented in this study are available on request from the corresponding author.

## Supplementary Materials

The following supporting information can be downloaded at https://www.mdpi.com/article/10.3390/jcm14041280/s1, Table S1: Synopsis of the literature on *T. indotineae* cutaneous infections.

## Abbreviations

The following abbreviations are used in this manuscript:

HTLV1	Human T Leukocytes Virus
HIV	Human immunodeficiency virus
ITS	Internal transcribed spacer
WHO	World Health Organization
