# Supplementary material for: Cutaneous Infections Caused by Trichophyton indotineae: Case Series and Systematic Review
Source: J Clin Med. 2025 Feb 14;14(4):1280. doi: 10.3390/jcm14041280 (PMC11856467; doi:10.3390/jcm14041280)
Supplement: Supplementary file 1 [file jcm-14-01280-s001.zip › jcm-3465143-supplementary.pdf]

Supplementary Table S1. Synopsis of documented skin infections caused by *Trichophyton indotineae* included in this review.

AGE	GENDER	GEOGRAFIC ORIGIN	TRAVEL HISTORY	OTHER RELEVANT INFORMATION	MEDICAL HISTORY	AFFECTED SITES	DISEASE DURATION (months)	PREVIOUS TREATMENTS*	FINAL TREATMENT				FIRST AUTHOR, year and reference (COUNTRY^)
									drug(s)	dosage of the oral antifungal drug	duration of the oral therapy	Outcome	
28	F	Bangladesh	Patients were recent immigrants or born in Indian subcontinent and had travelled back to their birthplace in last years		None	trunk, arms	NR	O-TER 250 mg/d for 9 weeks: partial response with positive culture; GRI 500 mg x2/d for 4 weeks	ITR	200 mg/d	12	Improvement; relapse 5 months after stopping ITR	Dellière, 2022[9] (France)
47	F	India		Father of the next patient	Diabetes mellitus, psoriasis	groins, axillae	NR	O-TER 250 mg/d	ITR	200 mg/d	12	Remission; relapse 1 year later	Dellière, 2022[9] (France)
20	M	India		Son of the preceding patient	None	groins, trunk, buttocks, legs	NR	O-TER 250 mg/d for 12 weeks	ITR	200 mg/d	8	Remission	Dellière, 2022[9] (France)
46	M	Bangladesh			Diabetes mellitus, dyslipidemia	groins, buttock, thigh, arms, face	NR	T-CS; O-TER 250 mg/d for 8 weeks	ITR + T-bifonazole	200 mg/d	12	Remission	Dellière, 2022[9] (France)
44	F	Bangladesh			Diabetes mellitus, dyslipidemia	groins, axillae, trunk, limbs	NR	T-CS	O-TER + T-ciclopirox	250 mg/d	-	Lost to FU	Dellière, 2022[9] (France)
39	F	India			Chronic hepatitis B	trunk, arms	NR	T-CS	FLU + T-TER	200 mg/week	16	Improvement; relapse 1 year later	Dellière, 2022[9] (France)
57	M	Sri Lanka			Crohn's disease; psoriasis	groins, buttocks, knees, shoulders, neck	NR	T-bifonazole; O-TER 250 mg/d for 8 weeks + T-CS (for severe psoriasis): partial response	O-TER	250 mg/d	8	Improvement; relapse 1 year later	Dellière, 2022[9] (France)

28	M	Bangladesh		Initial misdiagnosis as psoriasis	Asthma HIV-negative; HTLV1-negative No ID; no diabetes; no hematological malignancy	abdomen, back, buttocks, thighs	NR	T-CS; T-TER + O-TER 250 mg/d for 8 weeks; T-econazole + GRI 500 mg x2/d for 9 weeks; T-TER + O-TER 250 mg/d for 13 weeks; T-bifonazole + O-TER 250 mg/d for 9 weeks	T-voriconazole 1% once a day for 24 weeks			Remission; relapse 6 months later with cure obtained after further use of T-voriconazole for 2 months	Gueneau, 2022[10] (France)
27	M	Vietnam	No recent travel history	No contact with animals	None	right leg	2	herbal medicine	ITR + T-ketoconazole for 2 weeks	200 mg/d	1	Almost complete remission	Ngo, 2022[11] (Vietnam)
71	M	India	India	Absence of similar skin lesions in close contacts	Liver enzyme elevation	face, trunk, lower extremities	12	T-CS; O-TER 250 mg/d for 1 week; FLU 150 mg/d for 2 weeks	FLU	150 mg/week	2	Pending FU	Posso- De Los Rios, 2022[12] (Canada)
27	M	India	Thailand		None	thighs, buttocks	12	T-clotrimazole for 1 week; T-CS for 4 weeks; O-TER 250 mg/d for 4 weeks	ITR	100 mg/d	4	Remission (persisting at 2-month FU)	Posso- De Los Rios, 2022[12] (Canada)
29	M	India		No recent travel history in all patients (in 3 travelling to India > 1 year prior to the infection onset)	None	lower abdomen, back, thighs, genitalia	3	T-CS/clotrimazole for 3 months	T-ketoconazole for 4 weeks	-	-	Lost to FU	Posso- De Los Rios, 2022[12] (Canada)
26	F	India			None	face, lower back, thighs, pubic area	18	T-TER for several weeks; T-CS + T-ciclopirox for 2 weeks, ITR 100 mg/d for 4 weeks	FLU	400 mg/week	12	Pending FU	Posso- De Los Rios, 2022[12] (Canada)
28	F	India	India		Hepatitis B carrier	arms, abdomen, thighs, pubic area, legs	6	Unspecified T- and O-antifungals in India	ITR + T-ketoconazole	200 mg/d	1	No response (pending FU)	Posso- De Los Rios, 2022[12] (Canada)
38	M	India			None	face, groins	24	T-TER, T-CS for several weeks; T-ketoconazole + O-TER 250 mg/d for 4 weeks	ITR	100 mg/d	1	Treatment stopped due to skin rash (pending FU)	Posso- De Los Rios, 2022[12] (Canada)
78	F	India			Diabetes mellitus, hypertension,	feet, genitalia	6	T-CS for 4 months; T-antifungal; T-clotrimazole + T-CS	FLU	400 mg/week	12	Pending FU	Posso- De Los Rios, 2022[12] (Canada)

					dyslipidemia, osteoarthritis								
45	F	India			None	right forearm, left breast, proximal thighs	4	T-clotrimazole for 3 months	T-ciclopirox+ T-CS (declined ITR)	-	-	Pending FU	Posso- De Los Rios, 2022[12] (Canada)
28	F		No recent international travel history		None Pregnancy (at time of diagnosis)	neck, abdomen, pubic area, buttocks	6 ca.	O-TER (after delivery) for 2 weeks	ITR	NR	4	Remission (pending FU)	Caplan, 2023[13] (USA)
47	F	Bangladesh	Bangladesh (similar rashes noted in several family members)	Son and husband with similar rashes; eruption started while in Bangladesh	None	thighs, buttocks	6 ca.	T-antifungal and T-CS combination; T-CS and diphenydramine; T-clotrimazole; T-TER; O-TER for 4 weeks	GRI	NR	4	80% improvement (pending FU)	Caplan, 2023[13] (USA)
42	F	India		Family members without similar lesions; eruption probably started while in India		face, arms, trunk, groin, legs, toenails, fingernails	> 24	T-ketoconazole; FLU	O-TER + ciclopirox nail solution	250 mg/d	12	Remission	Crotti, 2023[14] (Italy)
33	F	Middle East	No recent travel history	Nurse in a dermatology department Family members without similar lesions Contact with cats	In the past, infantile eczema, rosacea, alopecia areata, irritable bowel syndrome, COVID-19 infection; bariatric sleeve gastrectomy	inner thighs, groins, buttocks, upper arms, face, anterior trunk	NR	O-TER 250 mg/d for 6 weeks; GRI 1 g/d for 6 weeks, ITR 200 mg x2/d for 4 weeks; T-TER, T-miconazole, fexofenadine	O-voriconazole	200 mg x2/d (after a loading dose of 400 mg x2/d for 2 days)	12	Remission	Dashti, 2023[15] (Kuwait)

27	M		No history of travel to a hyperendemic region for *T. indotineae*	Medical doctor; no contact with animals	HIV-negative; heterozygous p. N220fs*4 variant in the *CD36* gene	genitalia, scrotum, groins, pubic area, buttocks	60	T-antifungals, O-TER 250 mg/d for 6 months; ITR 100 mg x 2/d for 2 months; FLU 200 mg/d: effective with relapse after stopping treatment	O-resveratrol	NR	NR	Remission	Durdu, 2023[16] (Turkey)
25	F			No contact with animals	HIV-negative<br>No ID	thighs, buttocks	24	O-TER 250 mg/d for 6 months, T-TER; ITR 100 mg x2/d for 4 weeks: effective with relapse after stopping treatment; O-resveratrol	ITR	NR	8	Remission	Durdu, 2023[16] (Turkey)
32	M	India	No travel during past 4 years	A family member (father) with a similar rash in India; no contact with animals		buttocks, abdomen, left eyebrow	6	FLU 50 mg/d for 2 weeks; T-miconazole	ITR + T-bifonazole + T-ketoconazole (+O-glycyrrhizin 100 mg x2/d for 2 weeks)	200 mg/d	4	Remission	Jia, 2023[17] (China)
20	F	India	No travel during past 3 years			groins, thighs	NR	NR	ITR	200 mg/d	1	Remission	Jia, 2023[17] (China)
47	M	China	None		None	thighs, hips, neck	> 3	T-TER, ITR, T-CS + T-econazole; T-CS + T-ketoconazole; T-naftifine + T-ketoconazole;<br>O-TER 250 mg/d + T-ketoconazole for 10 days; O-TER + T-TER for 2 weeks: effective with prompt relapse after discontinuation	ITR + T-naftifine + T-ketoconazole	200 mg x2/d	3	Remission with recurrence after 2 weeks; new treatment with ITR for 5 weeks leading to remission without relapse at 3-month FU	Kong, 2023[18] (China)
21	F	Argentina	Mexico (in the last 18 months)		None<br>HIV-negative	buttocks, thighs	6	T-antifungals, FLU, O-TER	SUBA-ITR	100 mg/d	4	Remission	Messina, 2023[19] (Argentina)

32	F	India		Eruption started while in India	Breastfeeding	forearms	36	T-econazole for 9 months	T-ketoconazole for 3 months	-	-	No response<br>Lost to FU	Russo, 2023[20]<br>(Switzerland)
26	M	Afghanistan			None	groins, trunk, legs, arms	NR	NR	O-TER + T-ketoconazole	250 mg/d	16	Remission	Russo, 2023[20]<br>(Switzerland)
42	M			Other family member with dermatophytosis (not extensive)	None<br>HIV-negative<br>No ID	generalized (from face to legs including groins and genitalia)	6	O- and T-CS	ITR + T-luliconazole (+ oral antihistamine)	100 mg x2/d	8	Remission	Thakur, 2023[21]<br>(India)
17	F	Bangladesh				most of the body surface	12	O-TER 250 mg/d for 8 weeks	ITR	200 mg/d	12	Remission with localized new lesions 7 days after stopping ITR; new treatment with ITR 100 mg/d for 3 months with resolution (persisting at 6-month FU)	Villa-Gonzales, 2023[22]<br>(Spain)
26	M		Central and South America	During his travels, at times sharing of bedding and towels with others; eruption started while in Nicaragua; contact with cats and dogs	None<br>No ID	right leg, anterior aspect of the right thigh, groins, pubic area, left buttock	9	Multiple T-antifungals in Latin America<br>T-CS/clotrimazole; T-TER	ITR	200 mg once daily	8	Remission	Abdolrasouli, 2024[23]<br>(UK)
65	M	California	Europe		None<br>HIV-negative<br>Hemoglobin A1c = 6.2% (reference range ≤5.6%)	arms, legs, trunk, groins	NR	T-CS; T-clotrimazole; O-TER 250 mg/d for 2 weeks; GRI 500 mg/d for 2 weeks; O-TER 250 mg/d + O-prednisone (60 mg/d with tapering) + antihistamines; FLU 200 mg/d for 2 weeks	ITR + T-ketoconazole	200 mg x2/d (evening dose subsequently increased to 300 mg)	7	Remission (no recurrences at 8-month FU)	Bui, 2024[24]<br>(USA)

50s	F		Bangladesh	Unknown infection within household	No ID	forearms, feet, thighs, pubic area, buttocks, groins	6	T-CS; T-clotrimazole; T-ketoconazole; T-econazole; T-TER; GRI for 1 month; O-TER 250 mg/d for 6 weeks	FLU	150 mg/week	4	Remission	Caplan, 2024[25] (USA)**
20s	F		None	No infection within household	Pregnancy	abdomen, neck, pubic area, buttocks, inner thighs	13	T-TER; T-ketoconazole; O-TER 250 mg/d for 2 weeks	ITR	100 mg x 2/d	4	Remission	Caplan, 2024[25] (USA)
<10	M		Unknown	Unknown infection within household	No ID	abdomen, proximal legs	13	T-ciclopirox; T-clotrimazole; T-ketoconazole; T-TER; GRI for 2 months	ITR	NR	-	Lost to FU	Caplan, 2024[25] (USA)
40s	F		Bangladesh	Infection in family members	No ID	arms, axillae, buttocks, pubic area, inner thighs, upper legs	7	T-CS, T-clotrimazole; T-TER; T-ketoconazole; O-TER 250 mg/d for 4 weeks	GRI	5 mg/kg/d	8	Remission	Caplan, 2024[25] (USA)
40s	F		Bangladesh	No infection within household	Pregnancy	forearms, trunk, left axilla, left thigh	6	T-CS, T-clotrimazole; T-TER; T-ketoconazole	FLU	200 mg/week	12	Remission	Caplan, 2024[25] (USA)
20s	M		Bangladesh	Infection in a family member	No ID	left ear, left upper extremity, trunk, neck, groins	13	T-tacrolimus; T-penicillin; T-CS; emollient; antiseptic solution; T-naftifine; T-clotrimazole; T-econazole; T-ketoconazole; O-voriconazole; O-TER 250 mg/d for 4 weeks; ITR 100 mg x2/d for 8 weeks	ITR	200 mg x4/d	4	Remission	Caplan, 2024[25] (USA)
10s	M		Bangladesh	Infection in a family member	No ID	face, trunk, groins, buttocks	13	T-CS/econazole; T-clotrimazole; T-ketoconazole; T-econazole; T-TER; O-voriconazole; O-TER, GRI	ITR	100 mg x2/d	-	Improvement during treatment<br>Lost to FU	Caplan, 2024[25] (USA)

60s	M		Bangladesh	Infection in a family member	Lymphoma (untreated at diagnosis)	arms, thighs, buttocks, groins	3	T-ketoconazole; T-luliconazole	ITR	100 mg x2/d	-	Improvement during treatment	Caplan, 2024[25] (USA)
40s	M		Bangladesh	No infection within household	No ID	axillae, groins, buttocks	9	T-clotrimazole; T-ketoconazole; FLU	ITR	100 mg x2/d	6	Remission	Caplan, 2024[25] (USA)
30s	M		Bangladesh	Infection in family members	No ID	trunk, groins	10	T-CS; T-clotrimazole; T-miconazole; T-ketoconazole; O-TER 250 mg/d for 2 weeks; FLU 200 mg /week for 4 weeks (contraindications to ITR)	GRI	NR	-	Improvement during treatment after 2 months	Caplan, 2024[25] (USA)
20s	F		Bangladesh	Infection in family members in Bangladesh	No ID	legs, upper thighs, buttocks	42	T-CS, T-ketoconazole; O-TER 250 mg/d for 2 weeks	ITR	NR	-	ITR stopped due to gastrointestinal adverse effects<br>Lost to FU	Caplan, 2024[25] (USA)
60s	M				Therapy with ticagrelor (severe drug interaction with ITR)	buttocks	NR	O-TER 250 mg/d (multiple extended courses)	GRI + T-ciclopirox for the last 8 weeks	250 mg x2/d	20	Near complete resolution	Caplan, 2024[26] (USA)
22	M	Bangladesh				groins, hands, face, scalp	12	T-CS and unspecified T-antifungals and O-antifungal;<br>ITR for 4 weeks; O-TER for 8 weeks; ITR for 6 weeks	GRI<br>(GRI liquid was erroneously used topically instead of orally)	-	6	Improvement	Carroll, 2024[27] (Ireland)
25	M	Afghanistan	Pakistan		None	groins, thighs	15	T-TER; T-CS/clotrimazole; FLU 150 mg/week: improvement of truncal rash	ITR	NR	8	Significant improvement	Chua, 2024[28] (Australia)
33	F	Morocco		No contact with animals	Pregnancy	disseminated	6	O-TER 250 mg/d for 4 weeks	ITR (after delivery)	100 mg x2/d	6	Remission	Clemente Hernández, 2024[29] (Spain)

57	M		India	No infection within household	No ID	chest, back, abdomen, axillae, arms, groins	3	T-sertaconazole; T-TER; O-TER 250 mg x2/d for 8 weeks; ITR-SUBA 50 mg x2/d for 8 weeks; GRI 250 mg x2/d for 8 weeks	Posaconazole	300 mg/d	4	Remission	Fernandez-Gonzalez, 2024[30] (Spain)
43	M		India	No infection within household	No ID	groins, arms, buttocks	12	T-sertaconazole; T-TER; O-TER 250 mg x2/d for 8 weeks; ITR-SUBA 50 mg/d for 8 weeks; GRI 250 mgx2/d for 8 weeks	Posaconazole	300 mg/d	8	Remission with hyperpigmentation	Fernandez-Gonzalez, 2024[30] (Spain)
38	F		Bangladesh	No infection within household	No ID	groins, buttocks	6	T-clotrimazole; T-CS/clotrimazole; T-sertaconazole; O-TER 250 mg/d for 4 weeks; ITR 100 mg/d for 4 weeks	Posaconazole	300 mg/d	4	Remission with hyperpigmentation	Fernandez-Gonzalez, 2024[30] (Spain)
47	F		Bangladesh	No infection within household	No ID	face, groins, buttocks, axillae, back	6	T-CS/clotrimazole; O-TER 250 mg/d for 8 weeks; ITR 50 mg/d for 2 weeks (2 cycles)	Posaconazole	300 mg/d		Remission with hyperpigmentation	Fernandez-Gonzalez, 2024[30] (Spain)
40 (ca.)	F	Philippines	Dubai		Hypothyroidism No ID; no diabetes Steroid-induced rosacea	face	NR	T-ketoconazole; T- CS, T-neomycin	ITR	100 mg/d	4	Recurrence after O-doxycycline treatment for rosacea. Remission after use of ITR 400 mg/d for 1 week	Fukada, 2024[31] (Japan)
26	M	Israel	Jordan (recent travel)	Brother of the next patient	None	buttocks, groins, pubic area	15	O-TER 250 mg/ for 8 weeks ITR 200 mg/d for 8 weeks	Voriconazole	200 mg/d	12	Remission	Galili, 2024[32] (Israel)
28	M	Israel	Turkey (recent travel)	Brother of the preceding patient	Stage 4 Burkitt's lymphoma (receiving chemotherapy)	arms, buttocks, groins, knees, pubic area	15	O-TER 250 mg/ for 8 weeks ITR 200 mg/d for 8 weeks	Voriconazole	400 mg/d	8	Remission	Galili, 2024[32] (Israel)
15	M	Israel	None	Infection in a family member (brother) who	None	buttocks, groins, pubic area, thighs	12	O-TER 250 mg/ for 8 weeks	ITR	200 mg/d	8	Remission	Galili, 2024[32] (Israel)

				had visited Turkey									
51	F	India	India (recent travel)		None	axillae, buttocks, groins, limbs, pubic area, trunk	8		ITR	200 mg/d	4	Partial response<br>Lost to FU (patient returned to India)	Galili, 2024[32] (Israel)
45	M	India	India (recent travel)		None	buttocks, groins, limbs, neck, scalp, trunk	6	O-TER 250 mg/ for 8 weeks<br>ITR 200 mg/d for 8 weeks	ITR	400 mg/d	8	Remission	Galili, 2024[32] (Israel)
42	F	Vietnam				right axilla, right back, left calf	1,25	T-CS and salicylic acid; T-luliconazole for 6 weeks	ITR + T-lanoconazole	100 mg/d	4	Remission with recurrence 2 months later.<br>Retreatment with ITR + T-lanoconazole (lost to FU)	Mochizuki, 2024[33] (Japan)
36	F		India	Eruption started after traveling to India	None<br>HIV-negative<br>No ID	lower abdomen, thighs, genitalia, perineum	several	T-CS, FLU, T-TER; O-TER 250 mg/d for 3 weeks + T-TER + hydroxyzine; ITR 200 mg/d for 3 weeks with sparing use of T-CS (60% improvement); ITR 200 mg/d + T-ketoconazole + T-nystatin; ITR 200 mg x2/d (stopped after 1 week)	GRI	500 mg/d	8	NR	Smith, 2024[34] (USA)
NR	F	USA	South Asia	Intercourse in South Asia with a man with lesions on buttocks and genitalia; transmission to a new partner in the USA	None	buttocks, intergluteal cleft, pubic area	3 (ca.), from winter to spring	T-CS, T-econazole, O-prednisone, diphenhydramine, T-ketoconazole; O-TER 250 mg/d for 2 weeks; FLU 150 mg/week to 200 mg/d for >20 cumulative weeks	ITR	NR	1	Improvement with relapse after 6 weeks; new treatment with ITR 200 mg x2/d for 2 weeks with remission persisting at 3-month FU	Spivack, 2024[35] (USA)
25	M	Japan	None	Attending marine sports club where	None<br>No ID<br>HIV-negative;	groins, thighs	NR	T-butenafine + T- CS for 7 months; O-TER 125 mg/d for	ITR + T-ketoconazole	200 mg/d	4	Remission with relapse after 1 month (extension to the buttock and occipital region)	Takeuchi, 2024[36] (Japan)

				wetsuits were shared among members (some members with probable tinea cruris, subsequently treated)	HTLV1-negative			5 weeks; fosravuconazole 100 mg/d for 3 weeks				Retreatment for 12 weeks, with relapse in the occipital region 4 months later. Retreatment for 18 weeks (the patient was advised not to wear his favorite cap)	
25	F	India	India	Eruption started while in South India; no contact with animals	None<br>No ID	inframammary folds, pubic and suprapubic area, groins, inner upper thighs, buttocks	4		ITR + T-sertaconazole	200 mg/d	4	Remission	Tan, 2024[37]<br>(Singapore)
30	M	Turkey	Recently migrated from Turkey across several countries		None<br>No ID	right shoulder, inner arm, lower abdomen, groins, thighs	6	T-CS; T-antifungal agents	ITR	NR	6	Remission	Xu, 2024[38]<br>(USA)

* Previous treatments: duration and dose were specified only for systemic drugs (if information was available); when not specified, treatment was ineffective

^ Country: it refers to country of authors and where the patient was visited and the infection was diagnosed

**For reference 25, duration was defined as the interval between onset to confirmation

COVID-19= Coronavirus Disease 2019; F = female; FU = follow-up; HIV= human immunodeficiency virus; HTLV-1= human T-lymphotropic virus type-1; ID = immunodeficiency; M = male; NR = not reported; O- = oral; T- = topical; T-CS = topical corticosteroid; TER= terbinafine
